# Do Gut Microbes Taste?

**DOI:** 10.3390/nu13082581

**Published:** 2021-07-27

**Authors:** Ryan Leung, Mihai Covasa

**Affiliations:** 1 Department of Basic Medical Sciences, College of Osteopathic Medicine, Western University of Health Sciences, Pomona, CA 91766, USA; ryan.leung@westernu.edu; 2 Department of Health and Human Development, University of Suceava, 7200229 Suceava, Romania

**Keywords:** gut microbiota, taste function, microbiome, intestinal dysbiosis, appetite

## Abstract

Gut microbiota has emerged as a major metabolically active organ with critical functions in both health and disease. The trillions of microorganisms hosted by the gastrointestinal tract are involved in numerous physiological and metabolic processes including modulation of appetite and regulation of energy in the host spanning from periphery to the brain. Indeed, bacteria and their metabolic byproducts are working in concert with the host chemosensory signaling pathways to affect both short- and long-term ingestive behavior. Sensing of nutrients and taste by specialized G protein-coupled receptor cells is important in transmitting food-related signals, optimizing nutrition as well as in prevention and treatment of several diseases, notably obesity, diabetes and associated metabolic disorders. Further, bacteria metabolites interact with specialized receptors cells expressed by gut epithelium leading to taste and appetite response changes to nutrients. This review describes recent advances on the role of gut bacteria in taste perception and functions. It further discusses how intestinal dysbiosis characteristic of several pathological conditions may alter and modulate taste preference and food consumption via changes in taste receptor expression.

## 1. Introduction

The human body houses trillions of microbes along its surfaces and cavities. The gastrointestinal tract is the main location site that harbors approximately 4 × 10^13^ microorganisms that include not only bacteria but also fungi, archaea and virus-like particles [1]. Gut microbiota has been termed as the invisible metabolic organ for its important roles in host immunity, gut barrier integrity, metabolism, growth, fermentation of non-digestible complex carbohydrates, xenobiotic and drug metabolism, among other roles. These microbial communities, however, differ significantly along the gastrointestinal tract dependent on environmental variations in pH, oxygen exposure, and nutrient abundance [2]. Along with these environmental variations comes unique anatomical features that encourage distinct microbial colonization in proximity to taste receptors. As such, microbial colonies are topographically and strategically located near taste receptor cells in the mouth and intestine to facilitate optimal communication. Although many factors can influence the composition and function of gut microbiota, diet is the major modifier of intestinal microbial ecosystem. Likewise, microbes are also hypothesized to influence host eating behavior through a multitude of potential mechanisms involving gut–microbiota–brain axis. Among these, the evidence for microbial influence on taste perception and preference has been steadily increasing. Taste receptors are expressed in the gastrointestinal tract and they mediate nutrient assimilation via several mechanisms including secretion of gut peptides by enteroendocrine cells in response to stimulation by taste stimuli. The function of taste receptors in the detection of nutrients and the resultant effects on gustatory and digestive processes has been shown to implicate byproducts of gut microflora metabolism, such as short chain fatty acids, which can also serve as stimuli for taste receptors. Preservation of taste functions has been shown to depend on an intact gut microbiota [3] therefore, disruptions in the gut microbiota composition profile can results in taste changes. Taste, in this context, is not limited to gustatory function, but rather extends to the physiological detection of nutrients located throughout the gastrointestinal tract. Therefore, the focus of this review is to explore the supporting and growing evidence of how oral and intestinal microbial communities influence the host’s taste perception, and in turn, affect eating behavior. It further describes how taste receptors respond to changes in gut microbiota composition profile in disease conditions such as inflammatory bowel disease, chemotherapy and bariatric surgery.

## 2. Taste and Taste Receptors

Humans can recognize five primary distinct taste qualities: sweet, bitter, sour, salty and savory (or umami) and are also able to detect fat properties, although this is debatable [4,5]. This is accomplished via specialized taste receptor cells (TRCs) located within the taste buds that are distributed across tongue papillae and palate epithelium and epiglottis. The taste buds comprise approximately 50–100 taste cells that are embedded in fungiform, foliate and circumvallate papillae [6]. In response to a range of sapid stimuli, taste cells release neurotransmitters and other signaling molecules that convey taste information such as quality, intensity and hedonic value, to the rostral, gustatory portion of the nucleus tractus solitarius (NTS) that are then conveyed to several second order brain regions including the thalamus reaching the gustatory cortex, via facial, glossopharyngeal and vagus nerves [7]. The perception of sweet, umami and bitter tastes are mediated via G-coupled protein receptors (GPCRs) embedded in Type II taste cells. The sweet taste receptor is composed of TAS1R2 and TAS1R3 subunits while the bitter compounds are detected by 25 different taste receptors that belong to TAS2R family [8]. The umami receptor is a heterodimer composed of TAS1R1 and TAS1R3 subunits. Salty and sour tastes are presumably transduced via epithelial ion channels for sodium and hydrogen, respectively, on Type III cells, although less is known about these pathways [9]. Oral fat perception was long considered to solely be dependent on textural (trigeminal) and olfactory cues [10]. The identification of the fatty acid transporter CD36 as well as lipid specific GPCRs in taste bud cells have supported its gustatory qualities [11]. A recent study demonstrating chorda tympani nerve stimulation to linoleic acid specifically lends further credence to the notion of fat as a unique taste quality [12]. Once the tastant binds to the receptor it dissociates the heterotrimneric G protein (α-gustducin, Gβ3, and Gγ13) leading to increase in C-β2 (PLC-β2) activity. Activation of PLC-β2 results in production of diacylglycerol (DAG) and inositol 1,4,5-triphosphate (IP3) from phosphatidylinositol 4,5-bisphosphate. This activates inositol 1,4,5-trisphosphate receptor type 3 (IP3R3) receptors leading to release of calcium from intracellular stores and the gating of a transient receptor potential ion channel, TRPMP5 [13]. Activation of TRPM5 channel by calcium allows entry of Na+ that results in the depolarization of action potential, ATP release and convey the information to CNS. How primary afferent neurons respond to taste stimuli, particularly sweets, has been a matter of considerable debate with some suggesting that they respond best to sweet tastants from a range of taste stimuli while others suggest that they respond exclusively to sweet tastants. The “specialist” versus “generalist” responses or one taste quality versus “broadly tuned” has been shown to depend on the concentration of stimuli used, the type of the taste buds and the type of stimuli and whether single or in combination. While for sweet, bitter and umami, both specialist and broadly tuned responses have been shown, there is a dedicated labeled-line for the sour taste quality [4,14]. The brain gustotopic coding of taste quality responses is still unclear although it is more consistent with the concept of a distributed and wide brain network with highly variable responses between and within individuals [15,16]. This has important behavioral significance since overconsumption of taste stimuli such as sugars have been proposed as main contributors to obesity epidemic [17,18]. Several studies show that excess consumption of high sugar or high fat diet results in reduced response to sugar or fats as well as diminished neuronal responses to such stimuli [19,20,21,22,23] Therefore, deficits in sweet or fat responses can drive metabolic disorders such as obesity and diabetes. Among the numerous factors that are implicated in appetitive and consummatory behavior, the bacteria populating the digestive tract from the oral cavity to its distal segments play a critical role in modulating taste responses as reviewed in the next sections.

### Modulation of Taste Receptors by Nutrients

Sugars, minerals, organic acids, alkaloids and amino acids in foods act as chemical messengers by binding to their corresponding taste receptors [9]. This interaction represents one of the interfaces between internal and external milieus and transduces conscious gustatory perception through five known taste qualities: sweet, salty, bitter, sour, and umami [11]. Interestingly, the evidence has been growing for a sixth taste quality for fat and will be considered in this review [10]. The existence of these distinct taste qualities implies that each taste has a specific coding mechanism mediated by specialized taste receptors [11]. As such, the great variety of taste perception among individuals [24] leaves taste receptor expression and abundance as a potential variable dictating sensitivity and threshold levels. Due to its influence on eating behavior, genetic predisposition to taste perception, especially bitterness, has been a major focus of research over the last decades [25]. Increasingly, however, nongenetic factors like the gut and oral microbiota are being researched for their potential role in modulating receptor abundance and, in effect, taste perception. Perception can be defined as the intensity by which a signal is transduced and can be thought in terms of sensitivity and threshold.

Since identifying the chemical composition of foods is vital to homeostatic processes, it is not surprising that the same taste receptors of the tongue are also found in the gastrointestinal tract. These receptors then transduce nutrient signals into neuropeptide hormones, vagus nerve activation, or nutrient utilization [9]. Interestingly, the presence of intestinal hormones in isolated taste cells further points to the functionally similar role of lingual and intestinal nutrient sensors [26]. As such, it may be useful to consider both modalities when studying the modulatory effects of nongenetic factors like the microbiota. Current literature is generally consistent with the notion that when more receptors are expressed and activated, the signal to the brain is interpreted as more intense [27]. For example, Lipchock et al. [27] found that subjects who produced more bitter receptor mRNA perceived more bitterness when they tasted caffeine. This pattern of receptor abundance dictating perception has roots in observations of papilla and taste bud number on the tongue predicting taste intensity [28]. When extrapolating this to eating behavior, the general assumption is that the less sensitive to a taste (i.e., hyposensitive), the higher the consumption of that taste stimuli in order to reach the same stimulus [25]. This inverse correlation does not always apply, however, as other studies report the contrary claiming that a reduction in taste ability downregulates positive associations [29]. Links between tastes should also be noted as with supertasters: hypersensitivity to bitterness results in an aversion to vegetables, while a hypersensitivity to sugar results in increased hedonic response and higher consumption of sweet foods [25].

## 3. The Link between Gut Microbiome and Taste

The complexity of the gut microbiome such as richness and abundance have been used as a predictive factor of the host metabolic health. Diet and dietary habits are the main modifiers of the gut microbiome composition profile. Not surprisingly some determinants of obesity such as taste perceptions that influence changes in appetite regulation and energy metabolism have been linked with the gut microbiome. This relationship between taste and oral and intestinal microbiome is reviewed in the next sections.

### 3.1. Oral Microbiome and Taste

**Figure 1:**
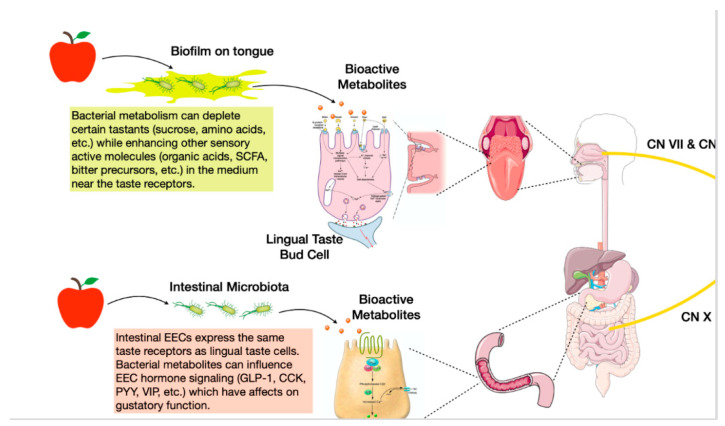
Microbes influence taste perception through biofilm and bioactive metabolites. The shape of the tongue dorsum forms a unique ecological site that encourages accumulation of a biological film composed of microorganisms. Bacteria can affect taste perception by acting as a physical barrier (top left) limiting access of taste molecules to taste receptors. Secondly, bacteria metabolism can modulate the concentration of peri-receptor tastants (top left), influencing taste receptor activation and taste sensitivity. Intestinal enteroendocrine cells (EEC) express the same receptors as lingual taste cells. Therefore, these potential mechanisms for bacterial taste alterations hold true for EEC as well (bottom left), allowing for further potential modulation of nutrient sensing.

The oral microbiome is one of the most stable and diverse ecosystems in the body due to the variety of niches present in the mouth and the abundance of exogenous nutrients during feeding and endogenous nutrients in salivary production [30,31]. Specifically, the tongue dorsum forms a unique ecological site that encourages accumulation of a biological film composed of saliva, oral debris, and microorganisms, an ideal habitat for microbial influence on taste perception [32]. Anatomically, the papillary structure of the tongue forms numerous depressions, effectively creating a reservoir for biofilm buildup and a large surface area for potential interactions. Although there is a continuous shedding of tongue epithelium, the tongue dorsum is hardly ever free from bacteria, such as *Staphylococci* and *Streptococci* [32]. Since the adherence of the tongue film is situated at the interface between tastants and taste receptors (localized in taste buds situated in the clefts of taste papillae), its consideration remains an important variable in taste perception studies [33,34]. Microbial tongue films can affect taste perception via two potential mechanisms of peri-receptor modulation: first, bacteria setting a physical barrier limiting access of taste molecules to taste receptors; second, bacterial metabolism modulating the concentration of peri-receptor tastants, thereby influencing taste receptor activation and taste sensitivity [33] (Figure 1).

For tastants to be perceived, they must first diffuse through the film coating the tongue and pass through the taste pore before binding to their corresponding receptor on taste buds [35]. Therefore, the microbial buildup of plaque likely blocks taste pores and prevents access to the receptors [36]. The bacterial load of tongue film can be measured by weight which was negatively correlated to taste sensitivity [33]. These findings are consistent with studies that seek to improve taste sensitivity by reducing tongue plaque through tongue brushing. For example, three months of tongue brushing in older adults significantly reduced tongue coating and improved the subjective and objective taste sensation of sweet, salty, sour and bitter, but not umami compounds [36]. This improvement was attributed to the removal of tongue plaque thus unblocking access to the taste pores.

#### Microbial Metabolites and Taste

Another possibility by which oral microbiome influences taste perception is through the consumption of tastants (sugars, amino acids) or synthesis of bioactive metabolites (organic acids, SCFA) before receptor interaction, ultimately inducing sensorial adaptation (Figure 1). Takahashi characterized the metabolic pathways of oral bacteria and how they may influence their environment. *Streptococcus (Firmicutes)*, *Actinomyces (Actinobacteria)*, and *Lactobacillus (Firmicutes)* species degrade carbohydrates into organic acids while *Prevotella (Bacteroidetes)* and *Porphyromonas (Bacteroidetes)* species break down proteins into amino acids and SCFAs [37]. While these characterizations were foundational for numerous gustatory studies, questions remain whether these bacterial metabolites are produced in significant enough concentrations to affect taste perception. A recent study by Gardner [38] used metabolomics to address this and provided supporting evidence that the net metabolic activity of oral microflora does in fact influence host taste perception. The authors measured consumption of the nutrient sucrose by the tongue biofilm by determining lactate/pyruvate ratios. A high lactate/pyruvate ratio was significantly associated with low-sensitivity sucrose perceivers compared to high-sensitivity perceivers, and these metabolic differences were hypothesized to be attributed to a higher abundance of efficient lactogenic microbes like *Streptococci* [38].

While bacterial metabolism may deplete sucrose, certain species can also enrich sensory-active molecules like acids in the medium near the taste receptors. This microbial production of acid does not induce a conscious sour sensation but rather increases sour detection thresholds through the sensory adaptation phenomenon. In this context, taste ability was reduced in the acutely hospitalized elderly, particularly those with high *Lactobacilli* growth [39]. By analogy, the presence of SCFAs was shown to be inversely associated with oral sensitivity to oleic acid [40]. In this latter study, fat detection hyposensitive subjects had an increased production of SCFAs which suggested the involvement of the oral microbes. Further studies have also indicated that certain bacterial phyla are positively correlated with an increase in taste sensitivity. For example, the presence of *Actinobacteria* and *Bacteroidetes* in tongue film are linked to an increase in taste sensitivity, especially to bitterness [33]. The mechanistic explanations are unclear; however, certain bacteria are known to produce secondary metabolites that act as precursors of some bitter acids. For instance, *Actinobacteria* produce phenols which can enhance the sensation of astringency and the bitter taste in food products [41]. When examining the differences between supertasters (high responsiveness to bitter PROP, ST) and non-tasters (low responsiveness to bitter PROP, NT) in the context of bacteria composition lining the tongue dorsum, Cattaneo et al. found that diversity did not differ significantly between ST and NT samples in terms of both taxonomic richness and evenness. However, at the level of single taxonomic units, they identified five bacterial genera whose relative abundances were significantly higher in ST than NT: Gram-positive *Actinomyces (Actinobacteria)*, *Oribacterium (Firmicutes)*, *Solobacterium (Firmicutes)* and *Catonella (Firmicutes);* and the Gram-negative *Campylobacter (Proteobacteria)* [42]. Taken together, these studies demonstrate how changes in the tongue microbial ecosystem can modulate taste perception (Table 1).

### 3.2. Intestinal Microbiome and Taste

Due to the high environmental variations in the distal gut, the density and composition of intestinal microbial communities differ greatly from those in the oral microbiota. Whereas oral microbes must endure periods of time without exogenous nutrients, gut microbes have a continual source of nutrients in the form of dietary fiber [38]. Although taste receptors are abundantly expressed in the oral cavity, they are also present in extra-oral tissues and are involved in many metabolic functions such as chemoreception, nutrient sensing, release of appetite hormones and other gastrointestinal functions. The mechanisms linking taste receptors and gut microbiota are not entirely known. Enteroendocrine cells (EEC) lining the GI tract are morphologically and strategically positioned in the intestinal epithelium to detect the presence of nutrients, as well as microbes and their metabolites. They express taste transduction molecules such as T1R2, T1R3 receptors and G-protein gustducin. The distribution of EECs varies based on the type, for instance, L-cells are present in high density in the ileum and colon. These areas contain the highest abundance of bacteria and therefore suggest an intimate relationship between bacteria and EECs [46] (Figure 1).

#### Intestinal Microbes and Taste Receptors

Taste receptors such as TAS2R are responsive to microbial-quorum sensing molecules and toxins by protecting against harmful microbes. For example, activation of TAS38 receptor increases the release of anti-microbial B-defensin and the hormone cholecystokinin. In response to changes in intestinal luminal environment, specialized EEC cells secrete a myriad of gut peptides such as CCK, GLP-1, GLP-2 and PYY. This complex regulatory process is mediated by a large family of cell-surface G-protein-coupled receptors (GPRC), expressed on the apical domain of EEC with a high degree of specificity for nutrient and taste sensing as well as microorganisms and toxic compounds. Interestingly, the GPCRs responsible for taste sensing and found in the lingual epithelium are also present in the intestine. For example, T1R2, T1R3, gustducin and GLP-1 are co-expressed in the same enteroendocrine cell. Therefore, the intestinal epithelium contains GPCR for detection of all nutrients, including fatty acids. Of interest is the fact that, indole, a tryptophan derivate and bacteria byproduct that modulates gut microbiota stimulate enteroendocrine cells to release GLP-1. It is known that GLP-1 receptors are expressed in gustatory neurons and are involved in transmission of taste signals, particularly sweet taste [47]. Furthermore, gut microbiota derived tryptophan metabolites reach the central nervous system and influence disease processes. For example, tryptophan undergoing microbial degradation is taken up by serotonergic neurons and glia and convert it to serotonin, a neurotransmitter that processes high order brain functions such as emotion, learning and memory. Compelling evidence also demonstrate the influence of gut microbes and tryptophan metabolites in the development of several neurological disorders such as Alzheimer’s disease, Parkinson’s disease and multiple sclerosis and indole derivatives have been used for neuroprotection [48]. Together, these studies show how gut bacteria can control maturation and function of CNS specialized cells through SCFA, vagal transit and metabolite production that cross the blood–brain barrier. This may also provide a signaling pathway to taste stimuli reaching the brain. Indeed, it has been reported that tryptophan acts as an agonist for bitter taste receptors and that indole interacts with TAS2R receptors. This suggests that other bacteria metabolites may act as ligands for taste receptor modulation leading to changes in phenotype [45].

The link between gut microbiota metabolites and enteroendocrine cells has been well documented [46]. Therefore, it is beyond the scope of this review to describe the mechanisms by which gut microbes-derived metabolites such as SCFA interact with EECs receptors. However, suffice to say that microbiota convert indigestible carbohydrates to SCFAs which signal to EE cells via free fatty acid receptors or activation of nuclear histone deacetylases (HDAC). Further, microbiota convert primary bile acids to secondary bile acids, which then signal to EE cells via the membrane G protein-coupled bile acid receptor (TGR5). Finally, structural components of the microbiota such as flagellin and bacterial lipopolysaccharide (LPS) activate Toll-like receptors (TLRs) with a role in maintaining gut barrier integrity, synthesis of antimicrobial peptides, inflammation and overall gut homeostasis. Aberrant TLRs activation results in dysbiosis and increased susceptibility to inflammatory and other metabolic disorders [1].

## 4. Microbes Modulate Taste Receptor Expression

In order to provide further insight into the complexities of human eating preference, a growing number of studies are seeking to characterize oral microbiota composition against the backdrop of taste sensitivity. A recent study by Cattaneo [25] found that generally, hyposensitivity towards a certain taste led to its increased consumption. In terms of relative abundance on tongue dorsum, Clostridia class was positively associated with protein/fat-rich diets and negatively associated with fiber intake. Proteobacteria phylum and Prevotella genus showed opposite associations and were more abundant in vegetable-rich diets. In a previous study, Cattaneo et al. [25] sought to differentiate oral microbiota compositions between supertasters (high PROP responsiveness, ST) and non-tasters (low PROP responsiveness, NT). In particular, the most responsive group (Supertasters) had an overrepresentation of five bacterial genera: the Gram-positive Actinomyces, Oribacterium, Solobacterium, and Catonella, and the Gram-negative Campylobacter (Table 1). In a cross-sectional study analyzing taste sensitivity in obese children, researchers found that subjects with a lower ability to perceive all taste qualities, especially bitter, had increased proportions of Bacteroidetes and Bacteroidia but decreased Proteobacteria [43]. A study that was centered around dental caries found that a decreased taste sensitivity for PROP was associated with increased risk for dental caries and higher *Streptococci Mutans* counts [44].

While these correlations between bacteria taxa and gustatory function do not yet have systematic explanations, a potential mechanism of receptor modulation by bacteria does arise from germ-free murine studies. In 2011, Swartz et al. found that germ-free mice had both a higher preference for sweets and a greater number of T1R2/3 sweet taste receptors in the proximal intestine compared to normal mice. While the absence of intestinal bacteria did not change lingual expression of T1R2/3, the increase of intestinal receptors seemed to have promoted long-term acceptance and preference for nutritive sweet stimuli. This phenomenon was primarily attributed to the post-oral nutrient feedback, reinforcing oral cues or taste associations, thus stimulating further consumption [3]. This study was important in that it described a compensatory mechanism for germ-free mice to consume more sugar in the absence of energy normally available from extraction by the gut microbiota. While this does not seem to influence gustatory sensitivity, the increase in gut T1R2/3 clearly affects the perception of sugar in the proximal intestine. However, in as much as these findings suggest the ability of microbes to modulate taste receptors, the ability of specific bacterial communities to restore the taste deficits in the germ-free animal model has not been tested.

This role of gut microbes is not limited to sweet tastants. In a similar study, germ-free mice showed an increased preference for intralipid emulsion that was associated with changes in lingual and proximal intestine fatty-acid receptors [29]. In absence of intestinal microbes necessary for optimal metabolism, the hypothesis was that the germ-free mice would exhibit a two-fold compensatory mechanism by increasing lipid consumption and decreasing post-ingestive feedback satiety signals. First, germ-free mice had increased lingual CD36 fat receptors which was associated with more fat consumption, contrary to other hyposensitivity eating behaviors [25]. Second, germ-free mice showed a decrease in intestinal fatty-acid GPRs and alterations in the abundance of enteroendocrine cells, ultimately resulting in a decreased hormonal satiety response and increased fat consumption [29]. These studies have established that the absence of microbes can lead to an altered receptor expression and potential gustatory changes. However, it is not clear what mechanisms or systems microbes utilize to maintain their influence on taste perception. The current literature indicates two likely pathways of modulation, the first one via the host immune system and the second via hormone secretion.

### 4.1. Microbes Influence Taste Perception through the Immune System

Taste buds face a unique challenge against pathogens being exposed to the oral cavity without a strong physical barrier. The presence of commensal microbes in proximity to taste and nutrient-sensing cells is complex and may also lead to an immune response in some instances. Indeed, microbial elements like lipopolysaccharide (LPS) and flagellin can induce inflammatory processes that have effects both locally and systemically. While these inflammatory expression patterns normally protect taste bud and nutrient-sensing cells from pathogens, it may also play a role in the modulating taste perception as well as the pathogenesis of taste dysfunctions [49].

**Figure 2:**
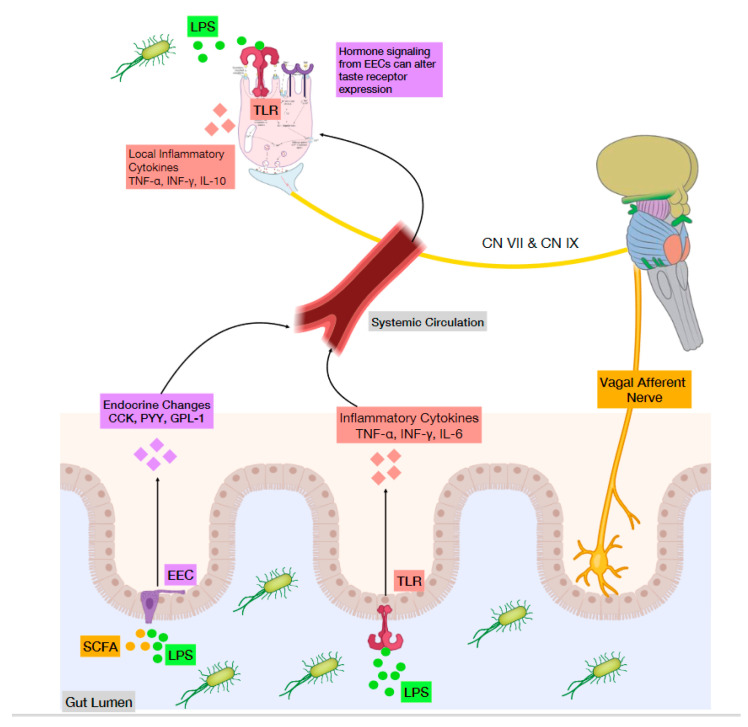
Microbes influence taste perception through the immune system and hormones. The microbial element, lipopolysaccharide (LPS), can induce inflammatory processes and hormone secretion that have local and systemic effects. Lingual LPS administration (top left) triggers a TLR response of inflammatory cytokine (TNF-α, INF-γ and IL-10) secretion and apoptosis of taste bud cells. LPS in the gut lumen (bottom center) interacts with TLRs to induces cytokine secretion (TNF-α, INF-γ and IL-6) into the blood stream that spreads to taste bud cells and alters tastant receptor expression and therefore taste thresholds. LPS also interacts with Enteroendocrine cells (EEC) (bottom left) stimulating release of gut hormones (CCK, PYY, GLP-1) that enter systemic circulation. These hormones are known to act as gustatory signaling molecules in taste bud cells. Microbial-induced taste responses (bottom right) might trigger a direct vagal mechanism to the brain.

Inflammation is initiated by the activation of Toll-like receptors (TLRs) by microbial pathogen-associated molecular patterns (PAMPs), as well as other inflammatory agents from damaged tissues or stress [50]. TLR signaling induces the expression of a variety of cytokines that then orchestrate an immune reaction (Figure 2). Wang et al. distinguished that in comparison to non-taste lingual epithelial cells, taste bud cells are enriched with several key inflammatory processing molecules. They showed how the LPS receptor TLR4, as well as other TLRs, are preferentially expressed in taste bud cells, suggesting a comparatively stronger response to PAMPs [49]. Interestingly, taste bud cells are not all uniform in their response to LPS. Upon close investigation, Feng et al. found that different subsets of taste cell types selectively produce specific cytokines such as tumor necrosis factor (TNF), interferon-γ (IFN-γ), and IL-10 [51]. In mouse taste buds, TNF is predominantly produced by T1R3-positive sweet/umami receptor cells [52] while IFN-γ is selectively expressed by a subset of type II cells and most type III cells [53]. The expression of the anti-inflammatory cytokine IL-10 was found exclusively in bitter receptor cells [54]. Similarly, a complex interplay also exists where these cytokines only affect specific types of taste cells with their associated receptors [51]. These studies show the existence of cell type-specific expression and transduction of cytokines taste type cells; however, the significance and their contributions to taste are still being studied.

Activation of the immune system can modulate taste perception by two means: acuity and sensitivity. The average turnover rate of taste cells is 8–12 days; therefore, a continuous supply of differentiated taste receptor cells is crucial for normal taste function [54]. A decrease in acuity can arise from a reduction in taste bud cell renewal and lifespan, an effect attributed to LPS-induced inflammation [53,55]. However, these effects on taste bud cells do not necessarily occur in isolation. Changes in sensitivity for specific tastes can arise simultaneously due to modification in taste receptor expression or through other means [56]. Several studies have further pointed to the immune system’s regulatory role in taste by measuring behavioral changes to specific cytokine deficiencies. For instance, TNF-knockout mice exhibit a decreased response to various bitter compounds, but not other tastes [52]. Additionally, IL-10-knock out mice have a reduced number of taste buds [54]. They also showcase an increased inflammatory response to LPS. These findings suggest that taste buds may use separate populations of taste receptor cells to modulate local inflammatory responses. Recent technological advancements of culturing taste organoids from progenitor cells promise a tool for broader manipulation and a deeper understanding of transduction mechanisms [57].

#### 4.1.1. Microbial-Induced Inflammation and Taste

Administration of LPS is an effective method for mimicking bacterial infection. Depending on where and how LPS is introduced, the immune response in taste buds can vary from hours to days later [56]. Therefore, it becomes imperative to distinguish either the location of bacteria presence or the mode of LPS delivery, lingual, systemic circulation, or via ingestion. Orally delivered LPS has been used to mimic exposure to bacterially contaminated food or water. Wang et al. [49] showed that acute lingual LPS administration triggered inflammatory cytokine release and apoptosis of taste bud cells. This resulted in abnormal cell turnover and a net loss of taste bud cells; however, it was unknown if this interfered with taste performance. Similarly, it is unclear if this phenomenon occurs during chronic low-grade inflammatory states, as seen in obesity [10]. In distinguishing how taste is affected by commensal lingual bacteria and not through LPS-induced inflammatory processes, Besnard et al. [58] sought to characterize what constitutes an “obese tongue”. It has been well documented that obese subjects have an impaired ability to detect lipids, which may lead to higher lipid consumption. They determined that obesity state and salivary LPS levels were both poor predictors for lipid sensitivity. They did, however, find specific bacterial compositions (increase in Bacteroidaceae family) in lipid non-tasters, irrespective of obesity status. These findings lend itself to the notion that lipid tasters may have an overall anti-inflammatory microenvironment while lipid non-tasters host pro-inflammatory bacteria. Interestingly, obesity amplified the phenotypic differences found between tasters and non-tasters, suggesting that this taste difference is mainly due to obesity once established.

Another method of measuring taste change has been through the systemic injection of LPS. For example, LPS injected intraperitoneally finds its way through mesenteric absorption and circulation to taste bud cells. This route represents an acute bacterial infection and has been shown to lead to the expression of inflammatory cytokine expression (TNF-α, INF-γ and IL-6) around circumvallate and foliate papillae. This LPS-induced inflammation was shown to inhibit the proliferation of taste progenitor cells and reduce the number of newly born cells entering taste buds. This also moderately shortened the average lifespan of mature taste bud cells [55]. Intraperitoneal injection of LPS has also been used to study its effect on Na+ transportation in taste buds, linked to salt taste perception. As such, Kumarhia et al. [59] found that LPS elicited an expression of the inflammatory cytokines TNF-α and IL-1β in taste bud cells. These cytokines had rapid effects attributed to modulation of channels (ENaC) responsible for Na+ transport: TNF-α reduced flux while IL-1β increased flux and was a more effective modulator. These results demonstrate that inflammation elicits swift changes in Na^+^ taste function, which may lead to heightened Na^+^ sensitivity during infection [59]. Interestingly, a previous study described how neutrophil recruitment to subcutaneous lingual injection of LPS might be responsible for observed sodium taste impairment. The neutrophil production of TNF-α and IL-1β has been suggested as possible mechanism; however, these cytokines were not measured in this study [60].

Lastly, the ingestion of LPS is a multifactorial vehicle that mimics consumption of harmful microbes and has been shown to affect taste. The timing of response following ingestion becomes a critical variable to consider. For instance, mice who ingested Gram-positive LPS bacteria exhibited a decrease in neural responses to sucrose during a single overnight period. A decrease in sucrose sensitivity was observed 7 days after injection, in parallel with decreased expression of sweet taste receptors T1R2+T1R3. These results did not occur in acute-, and lingual-treated LPS treated mice nor in TLR4 knockout mice, indicating that ingestion and proper immune responses were necessary to suppress sucrose response. Modulation of certain gut hormones known to affect peripheral taste function was listed as the potential mechanism of communication between the gut and taste bud cells [56]. In addition, gut permeability plays an important role since this method of LPS delivery introduces pathogenic material at intestinal interfaces.

While there are many protective mechanisms preventing LPS from crossing the gut barrier and entering systemic circulation, permeability has been shown to increase from gut dysbiosis induced by high-fat diets [61]. It has also been well documented that obese mice subjected to a high-fat diet exhibit a blunted ability to detect low concentrations of sweet solutions [62]. This effect was reversed following prebiotic supplementation that restored the eubiotic environment, demonstrating the role of gut microbiota on nutrient sensing [63]. Low-grade inflammation of adipose tissue might also be a factor influencing taste-driven reward behavior for foods rich in sugar and fat. As stated above, the communication between gut microbiota and other sensory systems such as taste require immune activation; however, this is not always the case. In fact, microbiota can elicit behavioral responses in absence of an immune response. For example, administration of the pathogen, *C. jejuni,* produced a rapid anxiety-like response in mice through a vagal pathway [64]. Since taste responses are also mediated by the vagus nerve, it is reasonable to hypothesize that microbial-induced taste responses might trigger a direct vagal mechanism to the brain.

#### 4.1.2. Microbes and Taste Sensing via TLRs

Toll-like receptors play a crucial role in sensing the intestinal microbes via recognition of pathogen-associated molecular patterns (MMAPs) that are derived from various microbes, triggering inflammatory and immune responses. Some TLR, such as TLR4 has been shown to influence anorexigenic signals [65]. For example, the pro-inflammatory lipopolysaccharides, acting mainly through activation of TLR4 receptors have also shown to increase GLP-1 secretion. Although some TLRs are expressed by EEC and administration of TLR agonists such as LPS or bacteria-derived lipoproteins stimulate secretion of gut hormones such as CCK and serotonin, TLR4 are expressed on the tongue gustatory papillae [49] and are involved in taste perception, food preference and intake [66] (Figure 2). However, only systemic and not lingual bacterial endotoxin mediated sweet taste functions via Tas1r2/3 receptors. Taken together, these findings demonstrate the intricate and intriguing mechanisms mediating taste signaling by bacteria. This has phenotypic correspondence in which germ-free mice consume more sucrose than conventional counterparts which was associated with increased intestinal T1R3 mRNA expression [3]. More recently, it has been shown that stimulation of the bitter taste receptors (TAS2Rs) present in gastrointestinal tract modulates enteroendocrine cell secretion and control food intake [67]. Although the exact mechanisms are not known, it is possible that microbial byproducts such as SCFAs acting on EEC cells and subsequent stimulation of gut peptides such as GLP-1, CCK and PYY might also contribute to this effect.

### 4.2. Microbes Influence Taste Perception through Hormones

Human taste bud cells secrete a number of diverse peptides as well as express cognate receptors [68]. These peptides are not only found in the gustatory system but rather play integral parts in regulating the body’s physiological response by acting on nervous or endocrine tissues. There are numerous studies demonstrating the gut microbiota’s ability to influence secretion of peptide hormones controlling appetite and energy regulation [46]. Thus, there is an emerging model of gut microbial influence on peripheral taste perception through these peptides and their autocrine, paracrine, and endocrine signaling. These large number of peptides expressed by taste bud cells are primarily studied in the context of metabolism, feeding, and satiety; however, at the lingual level, they may act to modulate adjacent taste cells and activate afferent nerve fibers [69] (Figure 2). The notion that these bioactive peptides play a role in processing taste information is supported by the expression of various cognate receptors by taste bud cells. While the precise functions of these peptides in taste buds are not fully understood, studies suggest that some act to modulate the responsiveness of the peripheral gustatory apparatus to certain taste stimuli [70].

Numerous studies over the years have sought to characterize the gustatory effects of these peptides [71] and showed their effect on taste qualities. For example, leptin decreases sensitivity to sweet [72] while endocannabinoids increase sensitivity to sweet [73]. Likewise, Glucagon Like Peptide-1 (GLP-1) increases sensitivity to sweet and decreases sensitivity to umami [74,75]. Cholecystokinin (CCK) may affect bitter taste [76]; Vasoactive Intestinal Polypeptide (VIP) modulates sweet, bitter and sour [77]; Peptide YY (PYY) increases responses to bitter and fat [78,79]; Neuropeptide Y affects bitter taste [80]; Oxytocin affects sweet and salty taste [81] and ghrelin increases responses to salty and sour [82]. While these peptides have been shown to locally affect taste perception, the question is whether circulating gastrointestinal peptides could influence taste buds in the same way. Indeed, the premise that peripheral taste functions are modulated by the metabolic state [68], supports this model of hormonal influence. Additionally, high concentrations of circulating leptin, along with TNF-a and insulin-like growth factor-1, have been found in individuals with increased taste responsiveness [83].

As stated above, the gut is the largest hormone-producing organ in the body [84] and houses the majority of the body’s microbiota. This complex interaction lends itself to the likelihood of microbes inducing downstream effects, consequently affecting an individual’s hormonal milieu and, in turn, their taste perception. Increasing evidence suggests that these gut microbes affect endocrine functions through two pathways: directly through the production of bioactive metabolites like SCFAs, and indirectly, as modulators of inflammatory responses, immune responses and hormonal secretion [85]. Importantly, SCFAs are directly implicated in the release of hormones and neuropeptides, such as GLP-1 and PYY from intestinal enteroendocrine cells [86,87]. For example, intestinal infusion of *E. coli* proteins also leads to an increase in plasma PYY and GLP-1 levels [88]. Further, ninety-five percent of SCFAs produced in the gut are represented by acetate, propionate and butyrate [89] and activate FFAR2 and FFAR3 receptors expressed in EECs with different potency or act via histone deacetylase (HDAC) inhibition. Both receptors activate Gα_i/o_ signaling; however, FFAR2 also signals Gα_q/11_ to release intracellular calcium leading to secretion of gut hormones [90] Recently, Shackley and colleagues reported upregulation of the umami taste receptor subunit TAS1R1 following exposure of EECs to SCFAs in STC-1 cells and murine intestinal organoids models. This data demonstrate how SCFAs can induce remodeling of GPCR gustatory signaling system [91].

The model of the gut microbiota as a stimulator of the immune system intersects with its role in hormone regulation (Figure 2). LPS is known to acutely increase circulating leptin levels in mice and rats via IL-1β [92]. In addition, mouse ingestion of LPS decreased taste responsiveness to sucrose, an effect attributed to changes in sweet enhancing hormones such as endocannabinoids, glucagon and GLP-1 [56]. Since leptin is derived from both gastric and adipose origin [93], the nutritional state should also be considered when studying associations between bacteria and hormone secretion. In conditions such as obesity, bacterial signals from the gut might compete with increased plasma levels of anorexigenic hormone signals like leptin [94] while prebiotic treatment improved leptin sensitivity [95]. Finally, changes in microbiota composition after bariatric surgery changed taste perception that may also be due to gut microbiota altering circulating hormone levels [84]. Taken together, these studies demonstrate that gut bacteria exert significant effects on circulating hormones which, in turn, influence taste.

## 5. Diet-Induced Changes in Gut Microbiota and Taste Perception

There are many known factors that drive food choices and habits, with taste considered as one of the main predictors [96]. This relationship between taste and food, however, can be described as bidirectional with dietary habits establishing taste perception as well. The previous sections described several mechanisms by which microbes influence taste perception. Since the role of diet in shaping oral and gut microbiota is widely recognized [25,97], diet-induced alterations in microbial signatures may contribute to gustatory changes.

Popular nutrition advice often claims that one can “retrain taste buds” by adhering long enough to a diet low in sugar, salt and fat [98]. Indeed, a randomized control trial found that reduced dietary intake of simple sugars altered subjects’ perceived sweetness intensity [99]. This study also found perceived pleasantness of these added sugar solutions to be unchanged, indicating that hedonic responses were not responsible for the change in taste perception [99]. This phenomenon of dietary modifications leading to taste perception changes is widely observed in murine studies as well, where exposure to high-fat diet decreases lingual sensitivity to fat [10]. The mechanisms by which oral and gut microbiota are reciprocally influenced are not yet fully understood; however, recent studies have found that the composition of oral cavity and stool bacteria overlap in 45% of subjects [100]. Therefore, one may hypothesize that dietary habits could affect these two microbial ecosystems in similar ways [25], and thus subsequent changes in peripheral taste and nutrient-sensing functions. In this context, potential parallels on taste and nutrient-sensing models can be drawn when discussing habitual diets.

Turner et al. [101] recently highlighted the gut microbiota’s likely involvement in altered nutrient sensing ability following regular consumption of artificial or intense sweetener (IS). Artificial sweeteners have been shown to have direct bacteriostatic effects on common gut microflora (*E. coli*) leading to dysbiosis (increased Firmicutes) [102]. Furthermore, receptor expression levels may change in response to this alteration in bacterial composition, resulting in altered metabolic functions like insulin resistance and obesity [101,103,104]. The gut microbiome’s role in these IS-consuming metabolic conditions is further supported by studies with fecal microbial transplant. Importantly, IS-induced glucose intolerance was fully transferable to germ-free mice and was shown to be eliminated through antibiotic treatment [105].

Another indication that the habitual consumption of certain foods leads to changes in gut microbiota and nutrient-sensing ability is evident from studies on consumption of inulin, a dietary fiber found in plants. A recent murine study by Weninger et al. [106] found significant improvement of small intestinal nutrient-sensing after 6 weeks of oligofructose (OFS) rich diet, a subgroup of inulin. Specifically, OFS improved intestinal lipid-sensing mechanisms by increasing CD36 expression, which is known to mediate the lipid-induced release of GLP-1. This improvement was attributed to changes in the gut microbiota as transplant of microbiota reproduce these results [106]. The correlation between inulin consumption and peripheral taste function has yet to be determined. Interestingly, however, a 2-week inulin-rich vegetable diet was found to reduce desires for sweet, salty, and fatty foods, while also increasing hedonic attitudes toward some inulin-rich vegetables [107]. Sucrose detection threshold did not change during the intervention, but considering the Weninger et al. [106] study, fat detection may be another variable to study in this regard.

Eating disorders associated with taste responsiveness might lend itself to the influence of gut microbiota [108]. These modifications in taste signaling mechanisms theoretically could lead to increased consumption of food substrates preferred by specific microbes for survival [103]. Indeed, obesity is associated with lower responsiveness to sweet and fat, which may be attributed to specific oral and gut microbial signatures functioning through immune and hormonal responses as presented previously [58,63]. For example, children with obesity had lower counts of fungiform papilae compared to normal weight subjects, which was associated with different salivary bacterial alpha-diversity and a lower ability to correctly identify taste qualities [43]. Furthermore, several bacterial genera differ between individuals with different taste sensitivity independent of the nutritional status, and that oral bacteria such as Selenomonas could be biomarkers for excess adiposity, suggesting a direct link between taste and specific microbiota signature [109]. At the same time, individuals with anorexia nervosa exhibit impaired taste perception, namely with sweet, salty and umami tastes [110,111]. These impairments have been shown to improve with weight gain [112] and while the role of the gut microbiota in this disorder is still unclear, current evidence suggests it could be a potent therapeutic option [113].

## 6. Clinical Implication of Microbiota/Taste Interactions

### 6.1. Taste in Inflammatory Conditions

Inflammatory diseases such as bowel disease (IBD) are associated with alterations in taste sensitivity. For example, in a human IBD case-control study, taste sensitivity was significantly reduced in all tastes except for sour [114]. Similarly, Melis et al. [115] found the same reduction in taste sensitivity as well as a significant increase in sour perception for IBD patients. The exact mechanism for taste alteration is not clearly understood; however, the oral microbiome and its interaction with the salivary enzyme gustin CAVI is thought to be central [115]. Gustin CAVI is a zinc-dependent enzyme that regulates the pH balance of the saliva and its disruption leads to a more acidic oral cavity environment [115]. Low salivary pH can lead to oral dysbiosis, and this has been observed in IBD patients who exhibit an increase in bacteria-derived acid metabolites [116], potentially contributing to sour perception alterations [115]. Gustin CAVI is also a trophic factor that promotes the development of taste buds [117] and its disruption may be a key factor in the overall decreased taste function of IBD patients [118]. Thus, it appears that intestinal dysbiosis precedes the onset of IBD [119] and this can cause intestinal malabsorption and zinc deficiencies [120]. Since gustin CAVI is zinc-dependent, the deficiency of this mineral due to dysbiosis may be linked to the inactivity of the gustin enzyme, changes in salivary pH, the progression of oral dysbiosis, and therefore taste alterations characteristic in IBD patients [115].

### 6.2. Taste and COVID-19

The SARS-CoV-2 spike glycoprotein (S) binds to angiotensin-converting enzyme-2 (ACE2) receptors that is abundantly expressed in the intestinal enterocytes’ brush border and colonic epithelial cells, supporting SARS-CoV-2 replication [121]. This results in local inflammation, disruption in resident microbiota and gut barrier dysfunction, thus decreasing secretion of antimicrobial peptides and facilitating bacterial metabolomes and byproducts to enter the circulation leading to systemic inflammation [122,123,124]. Several studies have shown that patients with COVID-19 have an altered microbiome characterized by an overall decline in microbial diversity, enrichment of opportunistic pathogens *Clostridium hathewayi, Actinomyces viscosus, Bacteroides nordii, Streptococcus, Rothia, Erysipelatoclostridium* and *Veillonella* along with significant depletion of beneficial commensals such as *Lachnospiraceae bacterium*, *Eubacterium rectale*, *Ruminococcus obeum*, *Fusicatenibacter*, *Eubacterium hallii*, *Anaerostipes*, *Agathobacter*, *Roseburia*, *Dorea formicigenerans*, *Clostridium butyricum*, *Clostridium leptum* and *Faecalibacterium prausnitzii* [125,126]. Some of these beneficial bacteria which includes butyric acid producing bacteria have been linked to reduced inflammation. Further, the probiotic bacteria, *Lactobacillus* and *Bifidobacterium* are also decreased in COVID-19 patients. It is not known whether changes in the gut microbiota environment due to COVID-19 are linked with taste changes in infected patients. It is widely known that a significant number of COVID-19 patients report taste changes, as well as changes in the overall oral sensitivity to commonly used condiments and spices. Recently, however, Doyle et al. demonstrated that ACE2 receptors are also present on a subpopulation of Type II cells, PLCβ2 positive, in taste buds, thus facilitating the entry of SARS-CoV-2 virus in the oral cavity [127]. Further, taste stem cell proliferation and turnover were reduced during the infection and lasted long after the onset of the infection. The authors hypothesized that the acute taste changes during COVID-19 are due to the replication of the virus and subsequent infection within taste buds, since sensory afferents of taste cranial nerves that carry gustatory signals to the brain do not express ACE2 receptors [128,129]. Oral microbiota is also disturbed in COVID-19 patients with significant diminution in species richness and marked differences in beta diversity. Specifically, there was a decrease in butyric acid-producing bacteria and an increase in lipopolysaccharide-producing bacteria. Therefore, changes in the microbiota composition profile are associated with aberrant inflammation that is present both in the oral cavity as well as in the lower gut of COVID-19 patients. In fact, abundance of certain gut bacteria such as *Coprobacillus*, *Clostridium ramosum* and *Clostridium hathewayi*, correlated with COVID-19 severity. Taste sensitivity is reduced in inflammatory conditions [126]; however, the contribution of systemic inflammation to taste changes in the oral cavity is not known. It is interesting to note that, Type II cells “taste” amino acids and ACE2 in the gut is involved in amino acid absorption [130]. Whether or not host ACE2 receptors co-localize with intestinal nutrients and taste receptors and how that might impact food choices remains to be investigated. Furthermore, oral microbial signatures can be used as a potential non-invasive diagnostic tool for COVID-19.

### 6.3. Taste, Chemotherapy, Drugs and Microbiome

Taste changes occur in up to 84% of patients undergoing chemotherapy treatments [131]. Recent studies report that taste disorders were more frequent in gastrointestinal than in breast cancer patients [132]. Patients who experience gastrointestinal symptoms during chemotherapy are also associated with an increased odd of having taste perception changes [133]. Cancer therapy is frequently associated with a disrupted microbiota, which may cause a release of inflammatory response ligands like LPS, bacterial DNA, and protein flagellin [134]. Previous studies have described how changes in gut microbiota are related to taste alterations in mice and this may be implicated in chemotherapy patients [3,29]. Wang et al. [135] have also proposed that the disruption of oral microbiota by chemotherapy agents could result in a local inflammatory response resulting in the observed taste changes. Indeed, oral mucositis is closely associated with alterations in taste and frequently reported in cancer patients undergoing chemotherapy [136]. While re-establishment of the microbiota in cancer patients has yet to be explored as a therapeutic treatment for taste dysfunctions, intensified nutritional counseling with taste and smell training has been shown to improve taste perception in these patients [132]. It should also be noted that many prescription and non-prescription drugs, including antibiotics, angiotensin-converting enzyme inhibitors, lipid-lowering agents, proton pump inhibitors, chemotherapy drugs, and metformin are known to affect taste as well as disrupt the gut microbiota. Whether these drugs can also impact the composition of the oral microbiota or its metabolites and induce taste changes is not known.

### 6.4. Taste, Bariatric Surgery and Microbiome

Several studies have shown that obesity is associated with taste changes, particularly a reduction in taste acuity and taste bud abundance. For example, hypothalamic and brainstem T1R3 and T2R116 taste receptors as well as signaling molecules such as Gα14 and TRPM5 were downregulated by obesity in mice [137,138]. In humans, loss of taste was associated with selection of high caloric foods [139]; however, the evidence linking taste responses and taste gene polymorphism is limited [140] Conversely, weight loss such in patients undergoing bariatric surgery results in rapid changes in taste which may be due to the overall reduction in inflammation and/or in response to physiological and metabolic changes due to the anatomical reconfiguration of the GI tract [141,142]. Indeed, bariatric procedures, such as Roux-en-Y gastric bypass (RYGB) and laparoscopic sleeve gastrectomy (LSG), results in significant changes in the anatomy, function, diet and intraluminal milieu of the gastrointestinal tract affecting the gut microbiota [143,144]. Gut microbiota plays a key role in pathogenesis of obesity and obesity has been characterized by a dysbiotic microbiota, with differences in both salivary and fecal microbiota composition profile and bacterially derived metabolites such as γ-amino butyric acid and butyrate between obese and normal weight individuals [126]. Changes in the gut microbiota composition profile are rapid, as early as one week after surgery, and are due to multiple factors including changes in diet, antibiotic treatment, anatomical reconfiguration of the gastrointestinal tract and weight loss. Bariatric surgery results in long-term weight loss, improvement of metabolic comorbidities like type 2 diabetes, increased satiety, decreased appetite and, interestingly, change in eating behavior [144]. Differences in microbiota composition profile, including salivary microbiota [145] between obese and lean individuals are well documented; however, how these changes impact taste is not clearly established. Similarly, the results from studies examining the effects of bariatric surgery on taste preference thus far are inconsistent. Some studies suggest changes in taste detection thresholds and acceptance to sweets. For example, RYGB patients had increased taste acuity for bitter and sour and increased threshold sensitivity for salt and sweets while in other studies patients showed high sour taste threshold or no difference in sensitivity thresholds for sweetness, bitterness or saltiness after RYGB compared with vertical sleeve gastrectomy (VSG) [146].

In a systematic review, Ahmed et al. [144] concluded that taste sensitivity to sweet and fatty stimuli appear to increase post-operatively bariatric surgery. Additionally, patients experience a reduced hedonic response to these stimuli. While the complex mechanisms leading to changes in eating patterns is still being investigated, some have hypothesized that an altered gut microbiota may play an indirect role [143]. As discussed previously, changes in the microbiota can alter circulating hormones levels, which might mediate the change in taste perception observed after bariatric surgery [68]. Increased levels of GLP-1 and PYY have been consistently demonstrated in rodent models of RYGB surgery [84]. Sanmiguel et al. [143] found that obese women who underwent bariatric surgery experienced reduced appetite and hedonic eating, which was associated with distinct gut microbial signatures. This study did not measure taste sensitivity, however. It is worth mentioning that hedonic responses are distinct from taste sensitivity and should be distinguished when discussing changes in eating behavior. Weight loss influencing eating behavior should also be considered as a confounding variable in RYGB studies. For example, Pepino et al. [147] found that women who experienced a 20% reduction in body weight post-operative RYGB reported a shift in sweetness palpability leading to a decrease in sweet food consumption. This change in eating behavior was not associated with changes in taste sensitivity, however, suggesting other unknown mechanisms. Finally, changes in microbiome post bariatric surgery have been associated with brain connectivity between precuneus and putamen regions of the brain that are involved in addictive behavior [148]. Therefore, the dramatic shift in the gut microbiota composition due to bariatric surgery may contribute to changes in taste and food cues in core regions of the brain similar to those seen in addiction behavior; however, this hypothesis needs to be investigated. Gustatory changes have also been identified in Type 2 diabetic patients who report decreased taste sensitivity for sweets, salty and sour stimuli [149] and are deficient in detecting fatty taste [150]. Furthermore, changes in lipid detection of diabetic patients have been associated with changes in the bacterial composition in the circumvallate papillae. As such, low lipid tasters had a greater bacterial diversity and proinflammatory profile with high *bacteroides/lactobacillus* ratio compared to high lipid tasters [150] that might explain reduction in fatty taste sensitivity. These changes in microbiota composition profile in high lipid tasters were associated with increases in bacterial metabolism involving catecholamine and ascorbate pathways.

## 7. Conclusions

Taste plays a significant role in food choices and is the most important driver of food consumption [151]. The increased hedonic value and motivation for palatable, energy-dense foods readily available in the current obesogenic food environment underscores the need for understanding the mechanisms by which enhanced motivation results in excess eating and seek new strategies to address dysfunctional eating behavior. Likewise, alterations in metabolic health can affect taste perception and preference. Taste is influenced by a myriad of factors including genetics, biological, physiological, metabolic, psychological and cultural. Although great strides have been made in understanding the role of gut microbiota on regulatory signaling controlling food intake and regulation of energy balance, including those involved in hedonic feeding such as taste, there remains much to learn. To better substantiate our knowledge of the complex and intricate interactions between the human host and gut microbes, the microbe to microbe interactions, their byproducts and effects on energy homeostasis, questions related to individuals’ microbial composition, host health status, taste polymorphism, genetics, internal and external influences, to name a few, must be answered. The differential regulation of the multiple microbial and antimicrobial compounds in both health and disease states and how these changes might impact consummatory behavior should be systematically dissected and study with deserved accuracy. These will lead to a better understanding of how to design prevention, diagnostic and treatment strategies in order to curb non-homeostatic excess eating, on one hand, and improve taste qualities in disease conditions, on the other hand.

## Figures and Tables

**Table 1:** Effects of bacteria on taste.

Microbes	Effect on Taste	Potential Mechanism	Reference
Staphylococci, Streptococci	Dampen sweet, salty, sour, and bitter tastes	Physical barrier through microbial tongue film	[26]
Streptococcus, Actinomyces, Lactobacillus		Degrade carbohydrates into organic acids	[31]
Prevotella, Porphyromonas		Degrade protein to amino acids and SCFAs	[31]
Streptococci	Alter sweet taste sensitivity	Degrade carbohydrate to lactate	[32]
Lactobacilli	Decrease sour taste sensitivity	Bacterial products raise sour detection thresholds	[33]
Actinobacteria and Bacteroidetes	Increase taste sensitivity, especially bitter	Bacteria produce secondary metabolites that act as precursors of some bitter acids	[27]
Actinomyces, Oribacterium, Solobacterium, Catonella, Campylobacter	Associated with high responsiveness to bitter		[36]
Clostridia	Associated with protein/fat-rich diets and negatively associated with fiber intake		[42]
Proteobacteria, Prevotella	Associated with vegetable-rich diets		[42]
Bacteroidetes, Bacterolidia	Associated with decreased perception of all tastes in obese children		[43]
Streptococci Mutans	Decreased sensitivity for bitter taste and increased risk of dental caries		[44]
Germ Free mice	High preference for sweet taste	Increased number of sweet taste receptors in proximal intestine	[3]
Germ Free mice	Increased preference for fat	Changes in lingual and intestinal fatty acid receptors; increased lipid consumption and decreased post-ingestive feedback satiety signals	[45]
